# Supplementary material for: Knowledge about research and facilitation of co-creation with children. Protocol for the article “scoping review of research about co-creation with children”
Source: PLoS One. 2024 Aug 9;19(8):e0307766. doi: 10.1371/journal.pone.0307766 (PMC11315329; doi:10.1371/journal.pone.0307766)
Supplement: S1 Checklist — (DOCX) [file pone.0307766.s001.docx]

**Checklist of the Preferred Reporting Items for Systematic Reviews and Meta-Analyses Protocols (PRISMA-P) statement adapted for a scoping review protocol.**

| **Section and topic** | **Item No** | **Checklist item** | **Page number in the manuscript** |
| --- | --- | --- | --- |
| **ADMINISTRATIVE INFORMATION** | | | |
| **Title:** | | | |
| Identification | 1a | Identify the report as a protocol of a scoping review | 1 |
| Update | 1b | If the protocol is for an update of a previous scoping review, identify as such | N/A* |
| Registration | 2 | If registered, provide the name of the registry (such as JBI) and registration number | N/A* |
| **Authors:** | | | |
| Contact | 3a | Provide name, institutional affiliation, e-mail address of all protocol authors; provide physical mailing address of corresponding author | 1 |
| Contributions | 3b | Describe contributions of protocol authors and identify the guarantor of the review | 14 |
| Amendments | 4 | If the protocol represents an amendment of a previously completed or published protocol, identify as such and list changes; otherwise, state plan for documenting important protocol amendments | N/A* |
| **Support:** |  |  |  |
| Sources | 5a | Indicate sources of financial or other support for the review | 14 |
| Sponsor | 5b | Provide name for the review funder and/or sponsor | N/A* |
| Role of sponsor or funder | 5c | Describe roles of funder(s), sponsor(s), and/or institution(s), if any, in developing the protocol | 14 |
| **INTRODUCTION** | | | |
| Rationale | 6 | Describe the rationale for the review in the context of what is already known | 3 |
| Objectives | 7 | Provide an explicit statement of the question(s) the review will address with reference to the inclusion/exclusion criteria | 5 |
| **METHODS** | | | |
| Eligibility criteria | 8 | Specify the study characteristics (such as PICO, study design, setting, time frame) and report characteristics (such as years considered, language, publication status) to be used as criteria for eligibility for the review | 7,8,9,10 |
| Information sources | 9 | Describe all intended information sources (such as electronic databases, contact with study authors, trial registers or other grey literature sources) with planned dates of coverage | 8 |
| Search strategy | 10 | Present draft of search strategy to be used for at least one electronic database, including planned limits, such that it could be repeated | 9 |
| **Study records:** | | | |
| Data management | 11a | Describe the mechanism(s) that will be used to manage records and data throughout the review | 9 |
| Selection process | 11b | State the process that will be used for selecting studies (such as two independent reviewers) through each phase of the review (that is, screening, eligibility and inclusion in meta-analysis) | 9,10 |
| Data collection process | 11c | Describe planned method of extracting data from reports (such as piloting forms, done independently, in duplicate), any processes for obtaining and confirming data from investigators | 10,11,12 |
| Data items | 12 | List and define all variables for which data will be sought (such as PICO items, funding sources), any pre-planned data assumptions and simplifications | 11,12 |
| Outcomes and prioritization | 13 | List and define all outcomes for which data will be sought, including prioritization of main and additional outcomes, with rationale | 11,12 |
| Risk of bias in individual studies | 14 | If this is to occur, describe anticipated methods for assessing risk of bias of individual studies, including whether this will be done at the outcome or study level, or both; state how this information will be used in data synthesis | N/A* |
| Data synthesis | 15a | Describe criteria under which study data will be presented | 12 |
|  | 15b | Describe the planned approach to how extracted data will be presented (such as figures, tables, evidence gaps maps) | 12 |
|  | 15c | Describe any proposed additional analyses (such as thematic analyses) | N/A* |
|  | 15d | If quantitative synthesis is not appropriate, describe the type of summary planned | N/A* |
| Meta-bias(es) | 16 | Specify any planned assessment of meta-bias(es) (such as publication bias across studies, selective reporting within studies) | N/A* |
| Confidence in cumulative evidence | 17 | If this is to occur, the method should be described | N/A* |

* N/A – Not applicable.
